# Quantifying the distance to criticality under subsampling

**DOI:** 10.1186/1471-2202-16-S1-O3

**Published:** 2015-12-18

**Authors:** Jens Wilting, Viola Priesemann

**Affiliations:** 1 Max-Planck-Institute for Dynamics and Self-Organization, D-37077 Göttingen, Germany; 2 Bernstein Center for Computational Neuroscience, University of Göttingen, D-37075 Göttingen, Germany

## 

Neuronal systems have been proposed to operate close to criticality. But how far from criticality are they precisely? We developed a novel method to determine the distance to criticality from data. Importantly, our method is reliable under subsampling, i.e. the experimental constraint that in many dynamical systems only a small fraction of all agents can be sampled. Thereby, our novel approach for the first time allows to determine the distance to criticality without bias from spiking activity in vivo, which in general is strongly subsampled.

In more detail, neuronal systems have been proposed to operate close to criticality, as power-law distributions of the avalanche size have been found for local field potentials from in vitro preparations [1], to human cortex [2]. Criticality is an attractive candidate state for neural dynamics, because in models criticality maximizes processing capacities [3]. However, it has been widely overlooked that criticality also comes with the risk of spontaneous runaway activity (epilepsy). Recent experiments suggest that spiking activity in rats, cats, and monkeys, is in a sub-critical regime, keeping a safety-margin from criticality [4]. Quantifying the precise distance to criticality may help to shed light on how the brain maximizes its information processing capacities without risking runaway activity.

**Figure 1:**
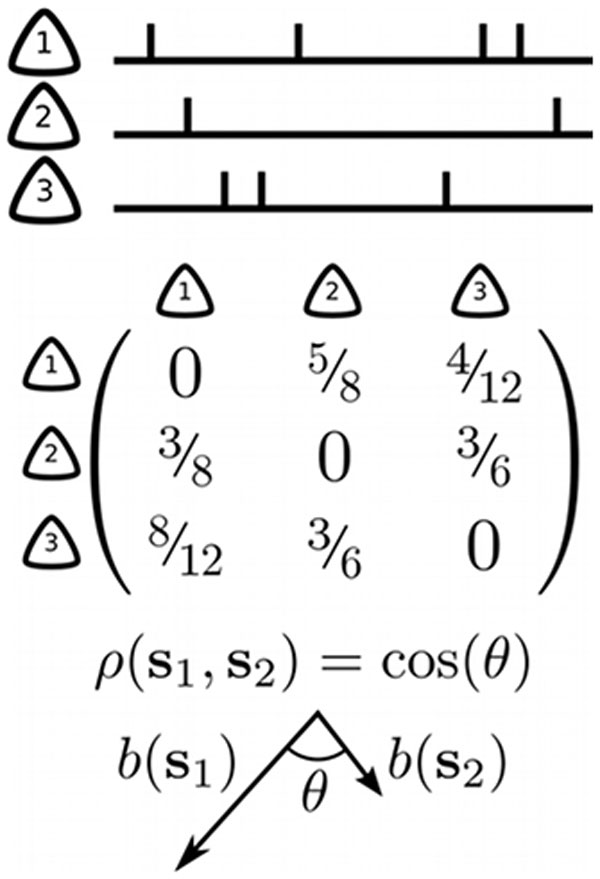
**Estimated branching ratio σ in dependence of sampled units n of a system of size N, for conventional (empty symbols) and our novel (full) measures in theory and models and in spike recordings**.

In neural systems, critical dynamics is usually studied in the context of branching processes with continuous drive [1], because they approximate well the functional propagation of spiking activity on the network [4]. The dynamics of branching processes are determined by the expected number of spikes σ in postsynaptic neurons triggered by a single spike, showing either stationary dynamics (sub-critical, σ < 1) or transient growth (super-critical, σ > 1); for σ = 1 branching processes are critical and generate the characteristic power law scaling. Methods to infer σ from fully sampled systems are well established, however, subsampling [5] resulted in strongly biased estimates (Fig 1., empty symbols). To overcome this bias, we derived a novel measure, based on a multistep linear regression. This measure for the first time allows to quantify the distance to criticality even under strong subsampling (Fig., full symbols). Our method generalizes to auto-regressive processes with both additive and multiplicative noise, making it widely applicable. We validated our method by applying subsampling to simulated branching processes with invasion, and to a generic integrate-and-fire model. After validation, we applied this method to highly parallel spike recordings from macaque prefrontal cortex, cat visual cortex, and rat hippocampus. These analysis indicated that spiking activity is clearly subcritical (0.97 < σ < 0.99; N = 10 experiments), and not critical.
